# Household context and child mortality in rural South Africa: the effects of birth spacing, shared mortality, household composition and socio-economic status

**DOI:** 10.1093/ije/dyt149

**Published:** 2013-08-02

**Authors:** Brian Houle, Alan Stein, Kathleen Kahn, Sangeetha Madhavan, Mark Collinson, Stephen M Tollman, Samuel J Clark

**Affiliations:** ^1^Institute of Behavioral Science (IBS), University of Colorado at Boulder, Boulder, CO, USA, ^2^MRC/Wits Rural Public Health and Health Transitions Research Unit (Agincourt), School of Public Health, Faculty of Health Sciences, University of the Witwatersrand, Johannesburg, South Africa, ^3^Section of Child and Adolescent Psychiatry, Department of Psychiatry, University of Oxford, Oxford, UK, ^4^Centre for Global Health Research, Umeå University, Umeå, Sweden, ^5^INDEPTH Network, Accra, Ghana, ^6^Department of African-American Studies, University of Maryland-College Park, College Park, MD, USA and ^7^Department of Sociology, University of Washington, Seattle, WA, USA

**Keywords:** Child mortality, socio-economic status, HIV, birth spacing, household, health and demographic surveillance system, rural, South Africa

## Abstract

**Background** Household characteristics are important influences on the risk of child death. However, little is known about this influence in HIV-endemic areas. We describe the effects of household characteristics on children’s risk of dying in rural South Africa.

**Methods** We use data describing the mortality of children younger than 5 years living in the Agincourt health and socio-demographic surveillance system study population in rural northeast South Africa during the period 1994–2008. Using discrete time event history analysis we estimate children’s probability of dying by child characteristics and household composition (other children and adults other than parents) (*N* = 924 818 child-months), and household socio-economic status (*N* = 501 732 child-months).

**Results** Children under 24 months of age whose subsequent sibling was born within 11 months experience increased odds of dying (OR 2.5; 95% CI 1.1–5.7). Children also experience increased odds of dying in the period 6 months (OR 2.1; 95% CI 1.2–3.6), 3–5 months (OR 3.0; 95% CI 1.5–5.9), and 2 months (OR 11.8; 95% CI 7.6–18.3) before another household child dies. The odds of dying remain high at the time of another child’s death (OR 11.7; 95% CI 6.3–21.7) and for the 2 months following (OR 4.0; 95% CI 1.9–8.6). Having a related but non-parent adult aged 20–59 years in the household reduces the odds (OR 0.6; 95% CI 0.5–0.8). There is an inverse relationship between a child’s odds of dying and household socio-economic status.

**Conclusions** This detailed household profile from a poor rural setting where HIV infection is endemic indicates that children are at high risk of dying when another child is very ill or has recently died. Short birth intervals and additional children in the household are further risk factors. Presence of a related adult is protective, as is higher socio-economic status. Such evidence can inform primary health care practice and facilitate targeting of community health worker efforts, especially when covering defined catchment areas.

## Introduction

Reducing child mortality is a central Millennium Development Goal, and although progress has been made in many regions, child mortality remains an important problem. The highest mortality rates continue to be in sub-Saharan Africa where in 2009 one child in eight died before his or her fifth birthday.^1^ Studies in sub-Saharan Africa have shown how various factors influence child mortality, including parental survival and breastfeeding and their interaction with HIV infection.^2^ The period following a mother’s death has been shown to be particularly hazardous in many studies,^3-7^ and it is also now evident that the risks for young children rise in the months before a mother’s death, as she becomes very unwell.^7^

However, less attention has been paid to other contextual risk factors, especially in light of the HIV pandemic.^8^ Studies of the household have focused on the economic burden of HIV, the role of older household members,^9^^,^^10^ fostering^11^^,^^12^ and orphanhood.^13^ We know from previous research in low- and middle-income countries that household composition can influence child mortality, but there has been little longitudinal research evaluating this influence in HIV-endemic areas.^14-16^

Household composition can affect child mortality in several ways. First, studies show that a variety of issues in relation to other young children can increase mortality risk. Short birth intervals before and after the birth of the index child increase mortality risk^17^^,^^18^ through short breastfeeding duration and maternal depletion. Similarly, having many children in the household increases family mortality risk through competition for limited resources, increased child malnutrition and greater risk of infectious diseases with fatal consequences.^19-21^ Lower per capita household resources and greater childcare responsibilities may compromise health care utilization.^22^ Further, there is a strong effect of the mortality of the previous sibling on the index child.^23^ The risk is also increased for infants of multiple births.^24^

Second, the presence of adults in the household can affect child mortality. Adults in the household provide resources for children and make care available. The effects of adults may vary by the presence or absence of the mother, and by how the adults are related to the child.^25^

Third, household socio-economic status (SES) can affect child mortality. Higher SES provides greater access to health services, adequate nutrition and sanitary conditions and other protective factors. The impact of SES follows a gradient: at the population level, the higher the SES of the household, the lower the mortality rate for children.^26^

Migration is a prevalent factor of life in the rural northeast of South Africa, a place that was preceded socio-politically by a labour migration system orchestrated by the state and key stakeholders in the country’s industrial development.^27^ Changes have followed the socio-political freedoms of the 1990s and increasingly young adults of both sexes migrate, as well as adults with spouses who are either left in the rural household or become migrants themselves.^28^ A proportion of adult migrants are also parents whose children have remained in rural areas. In the literature, the impact of this living arrangement on child mortality varies depending on a balance of positive or negative factors and the longevity of the migration system at a community level. The positive factors include benefits accrued from remittances^29^^,^^30^ and positive selection^31^ whereby migrants, especially labour migrants, tend to be healthier individuals from better-off households prior to migration. Negative factors include the impacts of social and biological disruption and consequent exposure to less healthy or less vigilant child care environments.^32^ The relationship between adult migration and child mortality is not static and changes over time because of the cumulative nature of the migration process improving conditions for children’s health.^33^

HIV can intensify or alter the effects of the household on child mortality. The presence of an adult with HIV may exacerbate household poverty,^34^ as may the loss or poor health of a caregiver for the children. The number of dependents in the household can also increase because of the need to support affected children, other vulnerable family members and orphans from other households. These additional demands may result in impaired food security, poor nutrition, compromised education and lowered income. Increasing diversity of household members may also lead to mortality differentials reflecting the degree of relatedness to the child.^35^

To inform policy and develop interventions it is necessary to understand the effects of household composition and relationships between household members on child mortality. By identifying key risk and protective factors in the household, we can clarify the timing and type of interventions necessary to improve child survival.

Our primary aim is to investigate the relationship between a young child’s risk of dying and the context provided by the child’s household. First, we examine the effects of other young children on the risk of dying. We include pre- and post-birth spacing (accounting for the index child’s age), multiple birth, the effects of resource competition with other children in the household and shared mortality risk among siblings. We explore the temporal relationship between children’s deaths within a household, relating the index child’s risk of dying to the amount of time before or after another child’s death. We also examine the effect of adults on the risk of dying, and whether the adult’s relationship to the child moderates these effects. Finally we examine the effect of relative household SES on the risk of dying.

We conduct this work in 27 contiguous villages in rural northeast South Africa, close to the border with Mozambique. Previously a so-called ethnic ‘bantustan’ under the Apartheid regime, the population comprises largely Xitsonga-speaking people of whom about a third originated from Mozambique as refugees during Mozambique’s civil war during the 1980s.

The area is poor and in need of infrastructure. Government-led development initiatives since democratic change in 1994 have been slow: some roads are tarred and all villages have access to electricity; however few households can afford electricity so reliance on fuel wood persists,^36^ water supply to village standpipes is erratic and sanitation rudimentary. People rely on a cash economy supplemented in important ways by state-sponsored, non-contributory social grants, particularly the old age pension^37^ and child support grants.^38^ Poorer households and those experiencing death of a breadwinner lack food security,^39^ with some 20–30% of children under 2 years of age stunted.^40^ Older women play a vital role in child care, feeding and schooling.^38^ Although primary education is virtually universal, quality is poor and progress often delayed. Employment opportunities in the area are few, with younger women increasingly migrating for work.^27^

A fifth of adults aged over 15 years (19.4%)^41^ and 4.4% of children aged 1–4 years^42^ are HIV-positive. Life expectancy has decreased markedly since the early 1990s and mortality has worsened in young and middle-aged adults as well as children.^43^ Primary health care services are free of charge; there are eight primary care facilities within the sub-district and three district hospitals 25–60 km away. As a result of the substantial risks to the population in this area, studies of mortality have been a focus of our ongoing work. This has included a range of analyses including the nature and temporal relationship of mothers’ and children’s deaths,^7^ but not the impact of other household factors.

## Methods

### Data

We use household census data from 1994–2008 for the population of approximately 82 000 people living in the rural Agincourt sub-district of the Bushbuckridge District, Mpumalanga Province, South Africa. Trained fieldworkers collect information annually by interviewing the most knowledgeable person in each household. Fieldworkers systematically collect data on all vital events (births, deaths), in/out migrations, nuptial events, household socio-economic indicators and other individual and household-level information.^44^ A verbal autopsy is conducted on all deaths; this involves physician assessment of a detailed interview with the closest caregiver of the deceased, to arrive at a probable cause of death including maternal causes.^44-46^ Robust quality control measures are in place, including checks at three field levels, a check at the main office and programmed computer checks, resulting in <1% missing data for non-censored individuals. We include all children aged 0–59 months with information on their mothers and household characteristics. Systematic information about the communities is not available from the annual census data.

The household definition includes temporary migrants who have been absent for more than 6 months of the previous year, provided that the respondent reports that the migrant intends to remain part of the rural household while s/he is away. That is, temporary migrants have another usual place of residence, generally closer to work, but are still considered part of the rural household and the study population.

An ‘absolute SES’ indicator was constructed from household asset surveys collected biannually since 2001. In constructing this indicator the aim was to keep it as simple as possible, retain an additive scale so that the final indicator scales in a cumulative sense and can be compared through time, and finally to recognize that assets fall into importantly different broad groups. To begin, each asset variable was coded with the same valence (i.e. increasing values correspond to greater SES) and effectively given equal weight by rescaling so that all values of a given asset variable fall within the range [0, 1]. Assets were then categorized into five broad groups: ‘modern assets’, ‘power supply’, ‘water and sanitation’, ‘quality of housing’ and ‘livestock assets’. For each household within each asset group, the rescaled asset values were summed and then rescaled again to yield a group-specific value in the range [0, 1]. For each household, these five group-specific scaled values were summed to yield an overall asset score that could fall in the range [0, 5]. The final overall score effectively gives equal weight to the five asset groupings, and within each group to each of the individual assets. A number of other more complex asset indicators were constructed and compared with one another and with the individual asset values. This indicator is highly correlated with all of the others and is at least or more correlated with the individual asset values as the others, and since it is far easier to calculate and explicate it was chosen for our final analysis.

### Statistical analysis

We use discrete time event history analysis to estimate children’s probability of dying by sex, age, mother’s cause of death, birth spacing, multiple birth, adults and children in the household, other child mortality and quintiles of household SES.^47^ We organize data as person-months, where a child is at risk of death from birth through each month s/he is observed up to and including when s/he dies or is censored. Variable values are assigned at the beginning of each person-month. Event history analysis accommodates two key features of longitudinal event histories: (i) both left- and right-censored observations; and (ii) time-varying covariates, which allow us to model household variables that change over time. Stillbirths were excluded from the analysis. Exploratory analyses separating neonatal deaths did not alter the substantive results (results not shown).

We use single or multi-level logistic regression to estimate the monthly probability of children dying. Multi-level models allow for correlation in mortality risks for children within the same household, which may reflect behavioural or socio-economic factors they share. To model this shared household environment, we included random intercepts for the household when this improved overall model fit, as indicated by the Bayesian Information Criterion (BIC).^48^ This variability is summarized using the median odds ratio. For randomly sampled children with the same covariate values, this term compares the child with the larger random intercept with the child with the smaller random intercept.^49^

Since SES was measured only from 2001, we use two models, each with a common set of covariates that include child sex, age, time period and maternal cause of death.

#### Model 1

Child and adult characteristics*:* pre-birth spacing was measured as number of months separating the births of the index child and their previous sibling, in ranges of 0–11, 12–23, 24–35, 36–47 and 48–59 months. Post-birth spacing was measured as the number of months separating the births of the index child and their subsequent sibling, in ranges of 0–11, 12–23 and 24–59 months (where the subsequent birth must have occurred before the beginning of the person-month), and depends on the index child’s age (0–23 or >23 months). Shared mortality risk was measured as the number of months up to and following another child death in the household. We also included an indicator of whether the previous sibling died before the conception of the index child. Resource competition was measured as the number of children living in the household at the time of data collection. A related household member was defined as anyone connected through the mother’s side of the family (but not the mother herself) and aged 5–19 or 20–59 years (there were not enough observations for those over age 60 to include in the model). Multiple birth classifies the index child as either a singleton or part of a multiple birth.

#### Model 2

SES characteristics*:* we classified household SES asset index scores into quintiles. In a given person-month, we applied the most recent SES measurement, starting in 2001. We modelled household SES separately because it was available only after 2001, which would shorten the historical scope of the data and reduce the sample size by over 50%. We tested a reduced model including household SES and child and adult characteristics: the substantive results were similar, suggesting that SES has an independent effect and does not explain our other findings.

## Results

Table 1 shows demographic characteristics for each estimation sample. The child/adult model includes years 1994–2008, whereas the SES model includes years 2001–08 (since SES was measured starting in 2001). The child/adult model is split into two periods up to and after 1998; 1998 represents the end of the rise in HIV prevalence in the population, which was followed by a high burden for an extended period of time afterwards.^50^ Differences between the estimation samples are thus mainly due to the different time periods included and the increasing burden of HIV/AIDS over time.

**Table 1:** Child and household demographics by model, accounting for child/adult characteristics and SES, Agincourt sub-district, South Africa, 1994–2008

	Model	
	Child/Adult	SES
	(*N* = 26 892)	(*N* = 7 550)
Child sex		
Male	13 375	3 783
Female	13 517	3 767
Mean age: years (SD)	2.12 (1.42)	2.34 (1.27)
Mean age by period: years (SD)		
1994–98	2.17 (1.41)	–
1999–2008	2.09 (1.42)	2.31 (1.27)
Mean age at death: months (SD)	13.71 (13.26)	20.41 (13.06)
Number of child deaths	659	319
Child deaths		
1994–98	116	–
1999–2008	543	319
Mean number of related family members (SD)^a^		
Aged 5–19 years	1.96 (1.48)	1.87 (1.38)
Aged 20–59 years	0.19 (0.55)	0.22 (0.60)
Mean number of children (0–59 months) per household (SD)	5.67 (3.65)	5.44 (3.54)

^a^Excluding mother and father.

The results from each model are presented in Tables 2 and 3 that contain odds ratios from the logistic regression of an index child’s death on the explanatory variables. The common covariates for each model show similar effects. A child’s odds of dying are high in the first 6 months of life and then decrease with age. The sex of the child was not significant in either of the models. A child’s odds of dying increased significantly in the years 1999–2008 relative to the period 1994–98 (Table 2). Finally, maternal death, and in particular death from HIV/TB, increases a child’s odds of dying.

**Table 2:** Logistic regression of child death by child/adult characteristics, Agincourt sub-district, South Africa (*N* = 924 818 child months)

	Odds ratio	95% CI	*P*-value
Child sex			
Male	1.043	(0.895–1.216)	0.593
Mother’s cause of death			
Alive	1.000	–	–
All causes except HIV/TB	5.123	(3.740–7.017)	<0.001
HIV/TB	7.537	(4.794–11.850)	<0.001
Time period			
1994–98	1.000	–	–
1999–2008	2.118	(1.726–2.598)	<0.001
Child age (months)			
0–6	1.000	–	–
7–23	0.457	(0.385–0.544)	<0.001
24–59	0.114	(0.089–0.145)	<0.001
Previous sibling death	2.641	(1.688–4.132)	<0.001
Length of previous birth interval			
No older siblings	1.000	–	–
0–1 year	1.352	(0.721–2.534)	0.347
1–2 year	0.702	(0.502–0.980)	0.038
2–3 year	0.795	(0.634–0.998)	0.048
3–4 year	0.813	(0.644–1.026)	0.081
4–5 year	0.886	(0.692–1.134)	0.336
Length of following birth interval			
Index child 12–23 months			
0–11 months	2.514	(1.102–5.732)	0.028
Index child 24–59 months			
0–11 months	1.523	(0.373–6.215)	0.558
12–23 months	0.318	(0.116–0.872)	0.026
24–59 months	0.476	(0.298–0.758)	0.002
Number of children 0–59 months in household			
No other children	1.000	–	–
1	1.15	(0.935–1.413)	0.185
2–3	1.172	(0.917–1.498)	0.205
4–6	1.569	(1.046–2.353)	0.029
6+	1.014	(0.409–2.513)	0.977
Months before/after another child’s death			
No child death/greater than −12/+12	1.000	–	–
−(6–12)	2.095	(1.231–3.566)	0.006
−(3–5)	3.009	(1.541–5.878)	0.001
−(1–2)	11.839	(7.641–18.343)	<0.001
0	11.671	(6.285–21.673)	<0.001
1–2	4.018	(1.879–8.589)	<0.001
3–5	1.688	(0.626–4.550)	0.301
6–12	1.92	(1.046–3.524)	0.035
Related household members (any)			
5–19 years	0.871	(0.705–1.076)	0.2
20–59 years	0.616	(0.456–0.833)	0.002
Multiple birth	1.765	(1.348–2.311)	<0.001

Unit of analysis is child-month. Explanatory variables defined at beginning of each month. Child death occurs anytime within month.

**Table 3:** Logistic regression of child death by household SES, Agincourt sub-district, South Africa (*N* = 501 732 child months)

	Odds ratio	95% CI	*P*-value
Child sex			
Male	1.002	(0.802–1.252)	0.988
Mother’s cause of death			
Alive	1.000	–	–
All causes except HIV/TB	9.828	(6.415–15.058)	<0.001
HIV/TB	13.167	(6.954–24.929)	<0.001
Child age (months)			
0–6	1.000	–	–
7–23	0.467	(0.335–0.650)	<0.001
24–59	0.11	(0.078–0.157)	<0.001
SES (quintiles)			
1^st^ (poorest)	1.000	–	–
2^nd^	0.665	(0.481–0.919)	0.014
3^rd^	0.532	(0.376–0.753)	<0.001
4^th^	0.681	(0.492–0.943)	0.021
5^th^ (least poor)	0.485	(0.338–0.698)	<0.001

	Parameter	95% CI	*P*-value

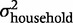	0.498	(0.140–1.774)	–
Median odds ratio	1.960	–	–

Unit of analysis is child-month. Explanatory variables defined at beginning of each month. Child death occurs anytime within month.

### Characteristics of other children in the household

Table 2 contains the odds ratios from the logistic regression of the index child’s death on the characteristics of other children aged 0–59 months in the household. A multi-level model including either the household or the mother as a random intercept did not improve overall model fit; we therefore used a single-level model.

### Pre/post birth spacing and multiple birth

**Figure 1:**
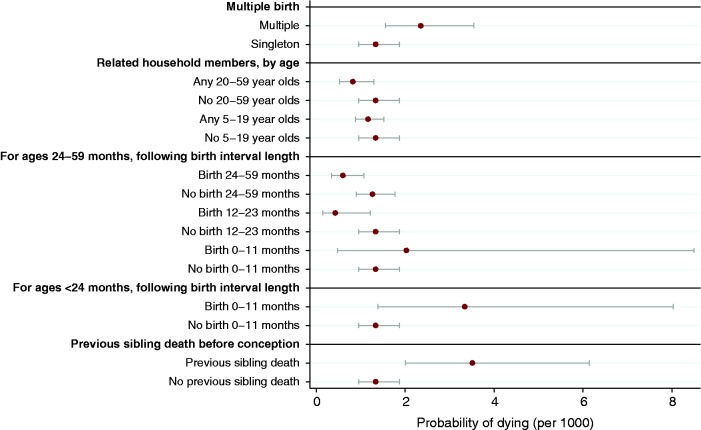
Monthly probability of child death by explanatory variables (indicator), Agincourt, South Africa, 1994–2008

For a child less than 24 months of age, having been born within 1 year of the previous child’s birth increases the probability of dying (OR 2.5; 95% CI 1.1–5.7), with a decline in mortality risk after this 1st year (Table 2 and Figure 1). Multiple birth increases the child’s probability of dying relative to singleton birth (OR 1.8; 95% CI 1.3–2.3) (Table 2 and Figure 1).

### Number of children

**Figure 2:**
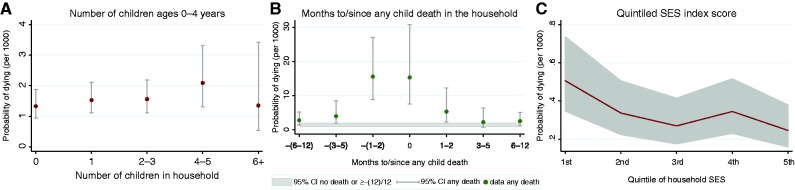
Monthly probability of child death by explanatory variables (ordinal and nominal), Agincourt, South Africa, 1994–2008

A large number of additional children in the household (related and unrelated) increases the probability that young children will die. Figure 2A shows that compared with a household with no other children, one with 4–5 additional children increases the index child’s probability of dying (OR 1.6; 95% CI 1.0–2.4). Breakdowns taking into account the sexes of the children, whether they were related or not and both sex and relationship showed similar effects (results not shown). This suggests that it is the overall number of other young children that increases the probability of dying and not their sex or relationship to the index child.

### Shared mortality risk

The odds ratios under the heading ‘Months before/after another child’s death’ in Table 2 show the effects of the number of months before/after any other child death in the household on the index child’s odds of dying. Figure 2B shows the monthly probabilities of dying, where at each time point the reference group is those children whose households did not have another child die during the same time span. A child’s probability of dying is high during the 12 months before the death of another child in the household (6–12 months before: OR 2.1; 95% CI 1.2–3.6; 3–5 months before: OR 3.0; 95% CI 1.5–5.9). Mortality risk rises dramatically 2 months or less (OR 11.8; 95% CI 7.6–18.3) before another child’s death, remains high at the time of another child’s death (OR 11.7; 95% CI 6.3–21.7) and for the first few months afterward (OR 4.0; 95% CI 1.9–8.6). Tabulations of children’s cause of death showed most cases to be undetermined. The variables remained significant even when we added a random intercept for the household, indicating that there is a relationship between timing of another child death in the household and the index child’s risk of dying.

### Characteristics of adults in the household

Table 2 contains the odds ratios from the logistic regression of the index child’s death on presence of related adults (aged 20–59 years) other than the parent in the household. The odds of dying decrease by 38% for children who have a related adult in the household compared with children who do not (Figure 1; OR 0.6; 95% CI 0.5–0.8).

Including unrelated older children and adults did not improve overall model fit (results not shown). Stratified regressions by the sex of older children and adults, and by sex of the index child, showed similar findings to those above (results not shown). There were not enough cases of related or unrelated adults over age 60 years to include in the model.

### Household socio-economic status

Table 3 contains the odds ratios from the multi-level logistic regression of a child’s death on the relative SES of the household. For this model, overall model fit was improved by including a random intercept for households. Figure 2C shows that relative to children living in households in the lowest SES quintile, each increase in relative household SES is associated with decreased odds of dying (except between the 3rd and 4th quintiles, where the odds of dying slightly increase). Compared with a child in the bottom (1st) quintile, a child in the top quintile (5th) has half the odds of dying (OR 0.5; 95% CI 0.3–0.7). The median odds ratio of 1.96 implies that when two children are simultaneously chosen at random from different households, the difference in their odds of dying will exceed 1.96 half the time.

## Discussion

Over a 15-year period in this rural area of northeast South Africa near the Mozambique border, in a population heavily burdened by HIV/AIDS, children were at elevated risk of dying in the year before another child in the household died, and this risk continued for several months after the other child died. This finding was robust to modelling unobserved heterogeneity at the household level, as well as to controlling for the survival status of the next-oldest sibling and the number of other children in the household. Mortality risks were high for very young children, especially in the first 6 months of life, and the risk increased when there were many other young children in the household; also, as previous research has found, children’s risk increased when post-birth intervals were short.^17^^,^^18^ The presence of a related adult in the household reduced mortality risk for children. Finally, even within this generally poor population, children in comparatively poorer households were more likely to die.

Previous research on death clustering of children in households has typically attempted to control for shared frailty in a household^51^ or operationalized the concept as a sequence of births and deaths within a family.^52^ Showing that child deaths are clustered temporally suggests further avenues of research to understand why mortality risk is clustered within households. Potentially testable causes include insufficient household income, inadequate sanitation, poor access to health care and other factors that are shared among children in the household and can change over time.

The presence of a related adult in the household reduces mortality risk for children, and may be particularly important in HIV-endemic areas. Given the poverty-inducing effects of HIV,^34^ extra adults can be an important way to reduce resource strain when funds are being directed to ill household members; they can also help provide care for children if the mother becomes very ill.^35^ The finding of a protective effect for related adults suggests that as household diversity increases (with inclusion of orphans from other households and other vulnerable extended family members),^53^ kinship-based effects on child mortality may become more important.^12^^,^^35^^,^^54^ The combination of low marriage rates, high non-marital childbearing, a history of labour migration, high unemployment and high HIV prevalence is increasing the complexity of household composition, which makes it difficult to categorize households into a small number of traditional types such as nuclear or extended. A child’s well-being depends on access to resources from adults other than biological parents, and it may be that investment in children is linked to genetic closeness.^55^ However, social anthropologists point to cultural preferences for ‘socially distributed childrearing’,^56^ in which a range of related adults are actively engaged in protecting children’s welfare. Evidence from other studies also indicates that proximity to kin is important to child well-being, including education, development and survival.^25^^,^^57^^,^^58^ The role of kin in protecting children is changing over time, and further research is needed to establish how this is occurring.

Labour migration is common in this population, and remittances can have beneficial effects on the welfare of children left behind in rural areas. Both male and female labour migrants contribute to the accumulation of assets that can improve the health of children.^41^ In the past, migration has negatively affected child health by compromising childcare. Before 2000, mothers migrated infrequently but when they did, there was a detrimental effect on under-five mortality.^26^ The balance of negative and positive factors has shifted over the years and the results now show a positive relation of mother’s migration to child mortality.^59^ Household resilience has improved, which has been caused by several factors. Remittances from women have increasingly contributed to household socio-economic status.^29^ The roll-out of government pensions has enabled children of migrants to stay with rural grandmothers who have more resources to support them. Child care grants have also added to household resilience in this way. Migration of the father was neutral to child mortality if he remained a household breadwinner, but detrimental if he was absent and not contributing to the household of origin,^26^ a finding independently corroborated by a study in rural Mozambique.^60^

This study confirms, in an HIV-endemic population, that mortality risk increases for children when post-birth intervals are short. Other studies have documented a similar pattern that is also influenced by the age of the index child.^17^^,^^61^ Our finding that mortality risk increases when there are many other young children in the household likewise echoes other studies, which have shown a fairly direct increase in child mortality with increasing numbers of children overall.^19^ Finally, we confirm the well-established finding^26^ that even within socio-economically disadvantaged areas, children are less likely to die in households with relatively higher SES.

The strong temporal association between multiple child deaths in the same household suggests common risks and pathways. Including a random intercept for the household did not attenuate the temporal association, indicating that this relationship is not explained by unobserved heterogeneity at the household level. Part of the explanation may be the easy transmission of infectious diseases among children in the same household, exposure to common and problematic child care practices or shared vulnerability to external shocks.

Over recent years, there has been a marked improvement in SES across households, as well as a compression of the distribution.^29^ This is increasingly well documented nationally,^62^ and can be attributed in large part to the extensive system of unconditional grants to pensioners (aged 60 years and over) and to those caring for children under 18 years.^54^^,^^63^^,^^64^ However, despite this improvement, mortality has (until recently) increased in this population because of the increasing burden of HIV/AIDS.^43^

We highlight the importance of household context, but how to apply this knowledge to public health practice is not self-evident. Yet our findings suggest stark, readily identifiable contextual indicators of adverse child health outcome. Community-based health workers (CHWs) are central to ‘primary health care re-engineering’, under way as new government policy in South Africa. In rural settings, CHWs are deployed to defined clinic catchment areas with oversight provided by nurses serving clinics nearby, an approach used in other countries of the region. Focusing on maternal and child health, the re-engineering initiative provides opportunity for CHWs to function in a nuanced way, identifying households and persons at risk, providing them with additional support and alerting key response groups (such as welfare and emergency services). Of course, this approach carries with it an imperative for skilled and supportive supervision of CHWs.

The strengths of this study include its use of detailed, longitudinal, systematic measurements of vital events, household membership and household SES over a 15-year period. However, there are a number of limitations of the study, including the fact that data are from a single geographical region in rural South Africa and so the extent of generalizability to other parts of Africa needs to be assessed. Whereas Agincourt has a somewhat lower infant mortality than parts of eastern and southern Africa, its adult female mortality is similar to that of eastern Africa,^65^ and its total fertility rate is similar to that recorded for southern Africa.^65^ We did not have information on the HIV status of either the child or other household members, and cause of death for many young children could not be classified through verbal autopsy. Finally, we used only household assets as a measure of SES; other factors, including remittances and pensions, could influence child well-being.

A key contribution of this study was to examine the effects of household context on child mortality over time in a population heavily burdened by HIV/AIDS. With accelerated roll-out of antiretroviral therapy and improvements in SES, settings such as this are undergoing rapid population and social transitions. This study has provided policy-relevant evidence on risk and protective factors, but household composition factors likely to affect child survival and well-being continue to change. Further research based on longitudinal data is needed to determine the ongoing impact of HIV on living arrangements and the changing role of kin in protecting the well-being of children.

## Funding

The MRC/Wits Rural Public Health and Health Transitions Research Unit, which has enabled the ongoing Agincourt health and socio-demographic surveillance system, has been funded by: the Wellcome Trust, UK (Grants 058893/Z/99/A, 069683/Z/02/Z and 085477/Z/08/Z); the Medical Research Council, University of the Witwatersrand, and Anglo-American Chairman’s Fund, South Africa; the Andrew W. Mellon Foundation; William and Flora Hewlett Foundation; and the National Institutes of Health (NIH), USA (Grant R24 AG032112). This study was supported by the National Institute of Child Health and Human Development (NICHD) of the National Institutes of Health (NIH) [grant numbers K01 HD057246 and R01 HD054511]. The content of the work presented here is solely the responsibility of the authors and does not necessarily represent the official views of the NIH.
